# Systematic Review of Literature on Eating Disorders During Pregnancy—Risk and Consequences for Mother and Child

**DOI:** 10.3389/fpsyt.2021.777529

**Published:** 2021-12-13

**Authors:** Małgorzata Janas-Kozik, Anna Żmijowska, Ida Zasada, Ireneusz Jelonek, Lena Cichoń, Andrzej Siwiec, Krzysztof M. Wilczyński

**Affiliations:** ^1^Department of Psychiatry and Psychotherapy of Developmental Age, Medical University of Silesia, Katowice, Poland; ^2^John Paul's II Pediatric Center, Sosnowiec, Poland

**Keywords:** pregorexia, anorexia, eating disorders, pregnancy, bulimia nervosa

## Abstract

**Background:** Eating disorders (ED) are a diagnostic category that includes several nosological units such as anorexia nervosa (AN), bulimia nervosa (BN), or binge eating disorder (BED). This category most often concerns women, while the peak incidence falls on the reproductive age. Therefore the issue of ED during pregnancy is an interesting topic. Due to the creation of unrealistic ideal of “desired,” slim figure both by the mass media and social media even during and right after gestation, more and more pregnant women introduce behaviours aimed at maintaining the “perfect” appearance. However in some cases it may have serious consequences for the health of both mother and child leading to the creation of the term “pregorexia” by the media to describe this issue.

**Aim:** The aim of this paper was to conduct a systematic review of the literature dealing with eating disorders in pregnant women, with particular emphasis on pregorexia.

**Method:** A systematic review of literature published within the last 5 years (2016–2021) in English or Polish and available through MEDLINE / PubMed, Google Scholar and Cochrane Library databases was conducted based on the previously assumed inclusion and exclusion criteria.

**Results:** Initially, 634 publications were obtained during the review, of which 55 papers were selected in the course of the title analysis. After further evaluation of abstracts, 28 papers were qualified for full text analysis. Ultimately, 10 papers were selected for the final analysis.

**Conclusions:** The issue of ED in pregnant women is a broad topic covering a heterogeneous group of women—both those with a previous history and those with the onset during pregnancy. The occurrence of ED symptoms during this period is associated with a high likelihood of negative consequences for both the mother and the child. The course of pregnancies and deliveries in these patients is more complicated. Therefore, it seems reasonable to develop a multidisciplinary screening strategy and standards of management and supervision over this group of patients.

## Introduction

Eating disorders (ED) are a diagnostic category that includes several nosological entities such as anorexia nervosa (AN), bulimia nervosa (BN), and binge eating disorder (BED). AN is characterised by an increased fear of weight gain, a disturbed body image, an introduction of dietary restrictions, or other weight loss behaviours. Bulimia nervosa is characterised by episodic occurrences of binge eating and the introduction of compensatory behaviours—most commonly purging to prevent weight gain. In the case of binge eating disorder, fewer compensatory strategies are observed than in the case of bulimia nervosa (1).

Statistically, women suffer from eating disorders much more often—Keski-Rahkonen et al. (2) indicate that the prevalence of anorexia nervosa among women in the European population is <1–4%, bulimia nervosa <1–2%, binge eating disorders 1–4%, while amongst men the prevalence of eating disorders is estimated at 0.3–0.7%. Eating disorders most often appear in young women (3) while they are still in adolescence or early adulthood—which coincides with the beginning of the reproductive period in women. Resultantly, the relationship between eating disorders and reproductive health remains a thought-provoking issue, especially in the context of sexual dysfunctions and menstrual disorders observed in women with ED (4). Amenorrhea is, according to the latest diagnostic criteria, one of the elements of the clinical picture of anorexia. Approximately 68–89% of female patients with AN confirm its occurrence for at least 3 months during the disease period. These types of disturbances in the monthly cycle are the result of limited caloric intake and/or excessive exercise, which lead to endocrine disruptions. Changes in the anatomy and physiology of the female reproductive system in the course of AN have led to the hypothesis in the literature that anorexia at least significantly hinders conception. However, recent studies indicate that the fertility of women diagnosed with AN does not differ from the general population. Furthermore, studies also indicate that women with anorexia nervosa tend to be of a lower average age upon becoming pregnant and are subject to the risk of unplanned pregnancy that is twice as high as that of the general population (5).

The period of pregnancy and puerperium is a period of immense changes taking place in the woman's body—including the greatest changes in body appearance since puberty (6). The time of pregnancy is also a period of greater sensitivity to negative self-perception, which contributes to the deterioration of self-esteem (7). For women with already diagnosed eating disorders, the period of pregnancy is particularly difficult (8), especially in the early stages of pregnancy. During this early period patients' body undergoes changes, yet they are not yet pronounced enough to clearly indicate pregnancy—this proves to be a particularly difficult moment for women with ED (9). Therefore, the impact of pregnancy on both the course of ED and the effect of ED on maternal and foetal well-being ought to be considered an issue of great importance. Sebastiani et al. indicate that the prevalence of eating disorders in pregnant women may reach 5–7.5% (6) and that the occurrence of these disorders has undoubtedly adverse effects on the course of pregnancy, childbirth and the condition of the child after birth, which becomes a significant interdisciplinary problem and is of equal interest to researchers in fields of psychiatry, obstetrics and neonatology.

Bannatyne, in a 2017 publication, indicates that a distinction can be made between eating disorders occurring before pregnancy, exacerbated during pregnancy or occurring only during pregnancy (10). It is estimated that on average 54% of women with a history of eating disorders reported improvement or even remission of ED symptoms during pregnancy (11). However, studies can be found that show the deterioration or appearance of new symptoms during this period, especially in the case of binge eating (12). The post-partum period presents as a particularly high-risk period for exacerbation and/or onset of symptoms and body image deterioration for women with and without a history of ED (13).

This topic is also an area of media interest—in 2008, on Fox news and The Early Show, journalists used the new term pregorexia to emphasise the emergence of a phenomenon involving restrictive behaviours such as limiting calorie intake and intensifying exercise amongst pregnant women in order to maintain a perfect figure during pregnancy and immediately after birth (14).

The aim of the present study was to carry out a systematic review of the literature on body image changes in pregnant women, the prevalence and course of eating disorders during pregnancy, and to attempt to gather current knowledge on the subject. In addition, it was sought to determine whether the media term pregorexia is medically reflected in case reports or diagnostic categories.

## Methods

The literature available electronically for the period 2016-2021 was reviewed in the course of the study. In this paper, the authors presented the current state of knowledge on eating disorders in pregnancy and therefore decided to include papers from the last 5 years in the review. The papers selected for the review were written in Polish and English. Articles were obtained from electronic databases: MEDLINE/PubMed, Google Scholar and Cochrane Library. The selected person-centred studies were chosen by searching the keywords “eating disorders,” “anorexia nervosa,” “bulimia nervosa,” “binge eating disorder,” “disordered eating,” “body image,” “pregnancy,” “pregorexia.” Each author searched for papers separately and these were then chosen in the three stage classification process. Initially, the authors searched the above- mentioned databases and, after evaluating the titles and preliminary analysis of the abstracts, selected the publications that were most relevant to the subject matter of the review. Selection was conducted according to the established inclusion criteria:

- Published between January 2016 and January 2021.
- Published in English or Polish,
- Published as part of journals (book excerpts, for example, and were excluded),
- Clear and comprehensive presentation of; the methodology (e.g., inclusion and exclusion criteria), demographic data of the participants and the process of diagnosis of the patients analysed.
- Good methodology was used including:
  ° reliable research tools, validated for the target population,
  ° clearly defined research hypotheses,
  ° satisfactory description of the statistical methods used.

**Figure 1:**
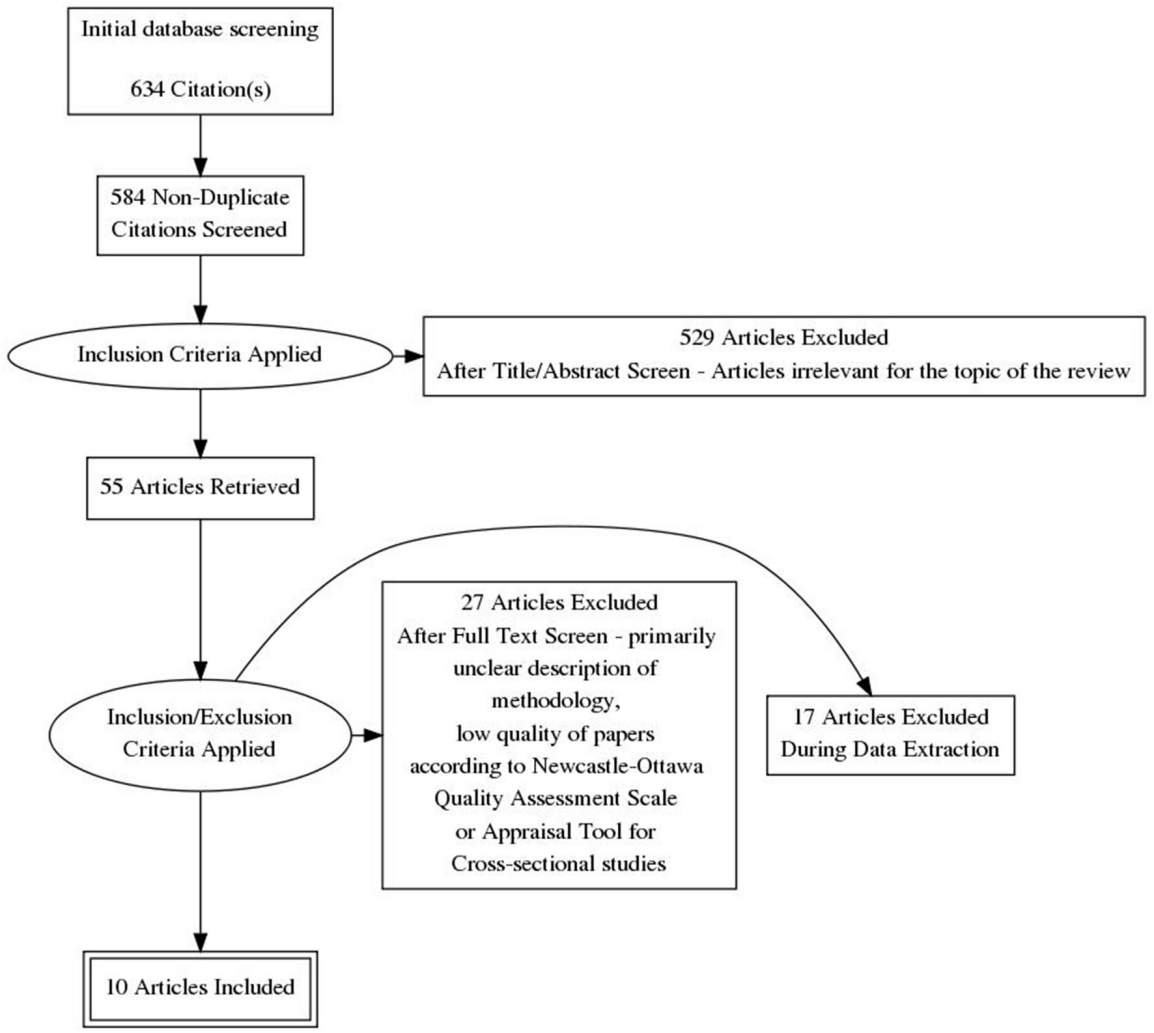
Procedure for qualifying publications for review.

Quality assessment of eligible cohort studies was conducted with utilisation of Newcastle-Ottawa Quality Assessment Scale while evaluation of cross-sectional studies was based on the Appraisal tool for Cross-Sectional Studies. The first phase of the review involved searching for publications, and analysing their relevance to the subject matter of the review, as based on the title of the paper. In the second stage, the abstracts were analysed for compliance with the study inclusion criteria. The publications, which were shortlisted by each author for further analysis were cross-checked against each other and, after exclusion of duplicates, were subjected to a preliminary full-text analysis aimed at assessing compliance with the assumed inclusion criteria. An analysis of the bibliography of the included works was carried out as well. Initially, 634 publications were obtained, out of which 55 papers were qualified in the course of title analysis. After further evaluation of abstracts, 28 papers were selected for full text analysis. The 10 papers found to be most consistent with the subject matter of the review were finally screened based on established inclusion and exclusion criteria. Figure 1 shows the procedure for qualifying publications for review.

## Results

Results are presented in the Table 1.

**Table 1:** Analysis of publications used in the review.

	**Title of the publication**	**References**	**Year of publication**	**Study group**	**Type of publication**	**Conclusions**
1	Maternal eating disorders and perinatal outcomes: a three-generation study in the Norwegian mother and child cohort study	Watson et al. (15)	2017	70,881 grandmother- mother-child triads (dataset 1- eating disorders during pregnancy) 52,348 grandmother- mother-child triads (dataset 2- eating disorder status during the lifetime)	Cohort study	Children of mothers with eating disorders during pregnancy presented numerous abnormalities after birth, and the course of these pregnancies was also abnormal.
2	Waking up every day in a body that is not yours: a qualitative research inquiry into the intersection between eating disorders and pregnancy	Claydon et al. (16)	2018	15 pregnant women	Qualitative Descriptive study - analysis of the interview and blogs/journals	The information obtained from the study is a source of help in prenatal and postnatal care for women with eating disorders.
3	Adolescent pregnancy and eating disorders: a minireview and case report	Harrison et al. (17)	2017		Case study of a 16-year-old girl diagnosed with atypical anorexia nervosa	Healthcare professionals should diagnose pregnant teens for symptoms of an eating disorder to ensure they receive timely treatment. In people with remission of ED symptoms, pregnancy is a key period in developing a treatment plan to maintain disease remission after delivery.
4	Association of maternal eating disorders with pregnancy and neonatal outcomes	Hirschberg et al. (18)	2019	7,542 pregnant women with ED, 1,225,321 pregnant women without ED	Cohort study	Women with active or prior eating disorders, regardless of subtype, are at increased risk of complications during pregnancy and in the newborns
5	Eating disorder symptoms pre- and postpartum	Petterson et al. (19)	2016	426 pregnant women, 345 in the postpartum period	Survey questionnaire	Study using the optimised and shortened Eating Disorder examination questionnaire EDE-Q, 5.3% of pregnant women and 12.8% of women in the postpartum period (6–8 months after delivery) suffered from eating disorders
6	Striving for the thin ideal post-pregnancy: a cross sectional study of intuitive eating in postpatum women	Lee et al. (20)	2019	419	Cross-sectional study	Survey questionnaire using: IES, BSQ, EAT 26 MBSRQ EPSD Most women in the postpartum period are at risk of negative unhealthy eating behaviours directed at
7	The relationship between perfectionism and body image with eating disorders in pregnancy	Kiani-Sheikhabadi et al. (21)	2019	200 pregnant women	Survey questionnaire	Survey questionnaire using: Eat 26, Bardone-Cone and rezaei perfectionism questionnaire. The authors point to a clear positive correlation between negative perfectionism (pursuing extremely high unrealistic goals) and the symptoms of ED in pregnancy
8	Body dissatisfaction and Fat Talk during pregnancy: predictors of distress	Dryer et al. (22)	2020	408 pregnant women	Survey questionnaire	Questionnaire study using: body part satisfaction scale (BPSS), PERCEIVED SOCIOCULTURAL PRESSURE SCALE (psps), internalisation of the thin ideal, fat talk questionnaire, Edinburgh postnatal depression scale, pregnancy related anxiety scale, eating attitude test eat 26. Body dissatisfaction causes symptoms of depression in pregnant women, ED and pregnancy related anxiety disorder (PABD). 9.3% had sufficiently high scores - the cutoff point for a screening diagnosis of an eating disorder
9	Prevalence and clinical characterisation of pregnant women with eating disorders	Bye et al. (8)	2019	545 pregnant women	Cross-sectional study	Depression, anxiety disorders, self-harm and suicide attempts were more common in pregnant women with eating disorders.
10	Presence of eating disorders and its relationship to anxiety and depression in pregnant women	dos Santos et al. (23)	2017	913 pregnant women (in the 2nd and 3rd trimester of pregnancy)	Cross-sectional and prospective study	Statistical significance was found between eating disorders and the risk of anxiety and depressive symptoms in pregnant women

## Discussion

The authors of the reviewed papers indicate a great variety in the clinical picture of eating disorders and in the level of severity of symptoms in a group of pregnant women. Claydon et al. (16) emphasise that pregnant women experiencing eating disorders can be divided into 3 groups: (1) those who improve during pregnancy and maintain improvement after childbirth, (2) those who improve only during pregnancy with secondary deterioration and symptoms intensification during the puerperium, and (3) women with active ED symptoms throughout pregnancy. Fogarty et al. (9) make further, more detailed distinction on the basis of the severity of ED symptoms during pregnancy. They distinguish between patients who (1) completely stop restrictive behaviour, (2) in whom a partial reduction in the severity of symptoms can be observed, (3) in whom symptoms remain at the same level as before pregnancy, and (4) a group of patients in whom symptoms worsen or possibly changes in the nature of symptoms develop—(e.g., from binge/purge type to binge-only type). For women who experience symptom reduction and health improvement in pregnancy, the desire to ensure the safety of the baby and a concern for the developing foetus constitute the protective factor (6). Claydon et al. (16) and Clark et al. (24) indicate that even foetal movements and kicks were protective factors against the negative body image. In their publication, Harrison et al. report that while eating disorder symptoms remit/weaken after pregnancy, their exacerbation can be observed after childbirth. Moreover, Lee et al. (20) indicate that changes in body appearance during pregnancy are more socially acceptable. What is more difficult to accept, however, is a woman's appearance after childbirth. During the post-partum period, women face pressure to return to their pre-pregnancy appearance and weight as quickly as possible, putting them at a greater risk of developing post-partum depression and anxiety disorders, especially in women with ED. These women are more likely to have problems adapting to their new role in life—being a mother—and show an increased risk of postnatal depression (19). dos Santos et al. demonstrated a statistically significant association between the presence of ED and the risk of anxiety disorders and depression (23). Bye et al. (8) indicate that psychiatric comorbidity is common in pregnant women with ED—these researchers also show that women with ED were more likely to suffer from depression and anxiety disorders. Moreover these women were more likely to have a history of self-harm and suicide attempts compared to women without a history of eating disorders.

At the same time, in patients with a history of eating disorders, the course of pregnancy, labour and puerperium is more often abnormal. In a 3-generation cohort study, Watson et al. (15) show that for mothers with eating disorders, the course of pregnancies was more often abnormal, and newborns presented numerous abnormalities after birth. Patients with BED faced an increased risk of gestational diabetes, pregnancy-induced hypertension and pre-eclampsia. Pregnant women with eating disorders were more likely to undergo caesarean section, induced labour, while the prolonged labour was more common. Their babies were born with lower.

APGAR scores, were more likely to require resuscitation and the incidence of perinatal death was more frequent. In addition, IUGR and smaller head circumference, microcephaly and low birth weight were more common. In their study, Watson et al. indicate that infants of mothers with AN had shorter body length at birth, low birth weight, while infants of mothers with BED achieved greater length at birth (LGA). The causes of this phenomenon are not entirely clear, but may include malnutrition or overfeeding, increased stress reactivity, persistent residual ED symptoms, or symptom recurrence (15). In their study, Mantel et al. indicate that all subtypes of eating disorders were associated wub an increased risk of preterm delivery and an ~2-fold increased risk of unrestrained purging. In contrast, women with present symptoms of anorexia nervosa showed a two-fold increased risk of anaemia during pregnancy and an increased risk of antenatal haemorrhage and instrumental delivery. In addition, newborns of mothers with ED were under an increased risk of microcephaly.

Arnold et al. (5) indicate a six-fold increase in perinatal mortality in newborns of mothers with ED. Bulik et al. (12) even discusses the risk cycle—mothers suffering from anorexia nervosa with low BMI give birth prematurely, which predisposes them to future onset of anorexia nervosa. Newborns exposed to perinatal complications are at a higher risk of developing symptoms of anorexia nervosa during adolescence and early adulthood (15).

The authors of this study did not encounter the term “pregorexia” in the analysed studies. None of the reviewed studies identified the group of women in whom the symptoms of eating disorders (i.e., restrictive behaviour—food restriction, intense exercise) were observed only during pregnancy and would disappear after delivery. Presently, the term “pregorexia” is not reflected in the current diagnostic categories.

Table 2 summarises possible complications in pregnant women with eating disorders and in their newborn children.

**Table 2:** Possible complications in pregnant women with ED and their newborns (5, 6, 15, 17, 18).

	**Abnormalities during pregnancy and delivery, gynaecological complications:**	**Complications in a child:**
Anorexia nervosa (AN)	Post-partum haemorrhage	Shorter birth length IUGR, SGA, low birth weight
Bulimia nervosa (BN)	Gestational diabetes, preeclampsia, more frequent abortions, PCOS, induced labour	Low APGAR score at 1 min
Binge eating disorder (BED)	Pregnancy-induced hypertension, gestational diabetes, preeclampsia, increased rate of miscarriages, longer duration of 1st and 2nd stages of labour PCOS	Longer birth length, LGA

Explanation of abbreviations.

IUGR, Intrauterine Growth Restriction; IUGR, Intrauterine Growth Restriction; SGA, small for gestational age; LGA, large for gestational age; PCOS, Polycystic ovary syndrome.

In description of the case of a 16-year-old pregnant girl with anorexia nervosa, Harrison et al. (16) draw attention to the small amount of research on eating disorders in underage pregnant women—the main focus of majority of studies is adult mothers, while research indicates that up to 11% of children worldwide are born to underage mothers, and up to 7.5% of pregnant women may have some form of eating disorder.

## Conclusions

The authors of the reviewed papers indicated that pregnant women with eating disorders constitute a heterogeneous group of patients. A distinction was made between women with ED onset before pregnancy and with ED onset during pregnancy. It was emphasised that pregnant women are sensitive and susceptible to external signals, which may result in them developing eating disorders, the symptoms of which persist beyond the pregnancy and the post-partum period.

However, the term “pregorexia,” which is not currently included in any classification, did not appear in the studies, nor are the criteria that this disorder could meet specified. Moreover, in some patients with ED prior to pregnancy, symptomatic improvement was observed during pregnancy, while in some cases, on the contrary—deterioration was observed in the perinatal period. A systematic review of publications on eating disorders in pregnancy from the last 5 years uniformly indicated the occurrence of numerous complications in pregnant women with ED for the mother (both somatic and psychological complications), for the course of labour and the newborn. Therefore, it appears reasonable to observe all pregnant patients for occurrence of eating disorders and to intensify the supervision of those women in whom they have been confirmed.

For pregnant women with active ED symptoms or a history of ED and the children they give birth to, interdisciplinary collaboration between specialists in gynaecology, psychiatry, neonatology and paediatrics is required. Therefore, it is deemed necessary to develop new screening methods or to disseminate existing ones in order to improve the detection of ED in pregnant women. There is currently very limited literature on eating disorders in pregnant women, especially in adolescents, indicating the need for additional research in this area.

## Data Availability Statement

The original contributions presented in the study are included in the article/supplementary material, further inquiries can be directed to the corresponding author.

## Author Contributions

All authors made equal contributions to the idea, review, and preparation of the manuscript.

## Conflict of Interest

The authors declare that the research was conducted in the absence of any commercial or financial relationships that could be construed as a potential conflict of interest.

## Publisher's Note

All claims expressed in this article are solely those of the authors and do not necessarily represent those of their affiliated organizations, or those of the publisher, the editors and the reviewers. Any product that may be evaluated in this article, or claim that may be made by its manufacturer, is not guaranteed or endorsed by the publisher.
